# Proteolysis of CD44 at the cell surface controls a downstream protease network

**DOI:** 10.3389/fmolb.2023.1026810

**Published:** 2023-02-17

**Authors:** Birte Wöhner, Wenjia Li, Sven Hey, Alice Drobny, Ludwig Werny, Christoph Becker-Pauly, Ralph Lucius, Friederike Zunke, Stefan Linder, Philipp Arnold

**Affiliations:** ^1^ Anatomical Institute, Christian-Albrechts-University Kiel, Kiel, Germany; ^2^ Institute of Functional and Clinical Anatomy, Friedrich-Alexander-University Erlangen-Nürnberg (FAU), Erlangen, Germany; ^3^ Institute for Medical Microbiology, Virology, and Hygiene, University Medical Center Eppendorf, Hamburg, Germany; ^4^ Department of Molecular Neurology, University Hospital Erlangen, Friedrich-Alexander-University Erlangen-Nürnberg (FAU), Erlangen, Germany; ^5^ Biochemical Institute, Christian-Albrechts-University Kiel, Kiel, Germany

**Keywords:** shedding, meprin β, ADAM10, MMP14, CD44, tumor spheroid, cell adhesion, MMP2

## Abstract

The cell surface receptor cluster of differentiation 44 (CD44) is the main hyaluronan receptor of the human body. At the cell surface, it can be proteolytically processed by different proteases and was shown to interact with different matrix metalloproteinases. Upon proteolytic processing of CD44 and generation of a C-terminal fragment (CTF), an intracellular domain (ICD) is released after intramembranous cleavage by the γ-secretase complex. This intracellular domain then translocates to the nucleus and induces transcriptional activation of target genes. In the past CD44 was identified as a risk gene for different tumor entities and a switch in CD44 isoform expression towards isoform CD44s associates with epithelial to mesenchymal transition (EMT) and cancer cell invasion. Here, we introduce meprin β as a new sheddase of CD44 and use a CRISPR/Cas9 approach to deplete CD44 and its sheddases ADAM10 and MMP14 in HeLa cells. We here identify a regulatory loop at the transcriptional level between ADAM10, CD44, MMP14 and MMP2. We show that this interplay is not only present in our cell model, but also across different human tissues as deduced from GTEx (Gene Tissue Expression) data. Furthermore, we identify a close relation between CD44 and MMP14 that is also reflected in functional assays for cell proliferation, spheroid formation, migration and adhesion.

## 1 Introduction

**FIGURE 1:**
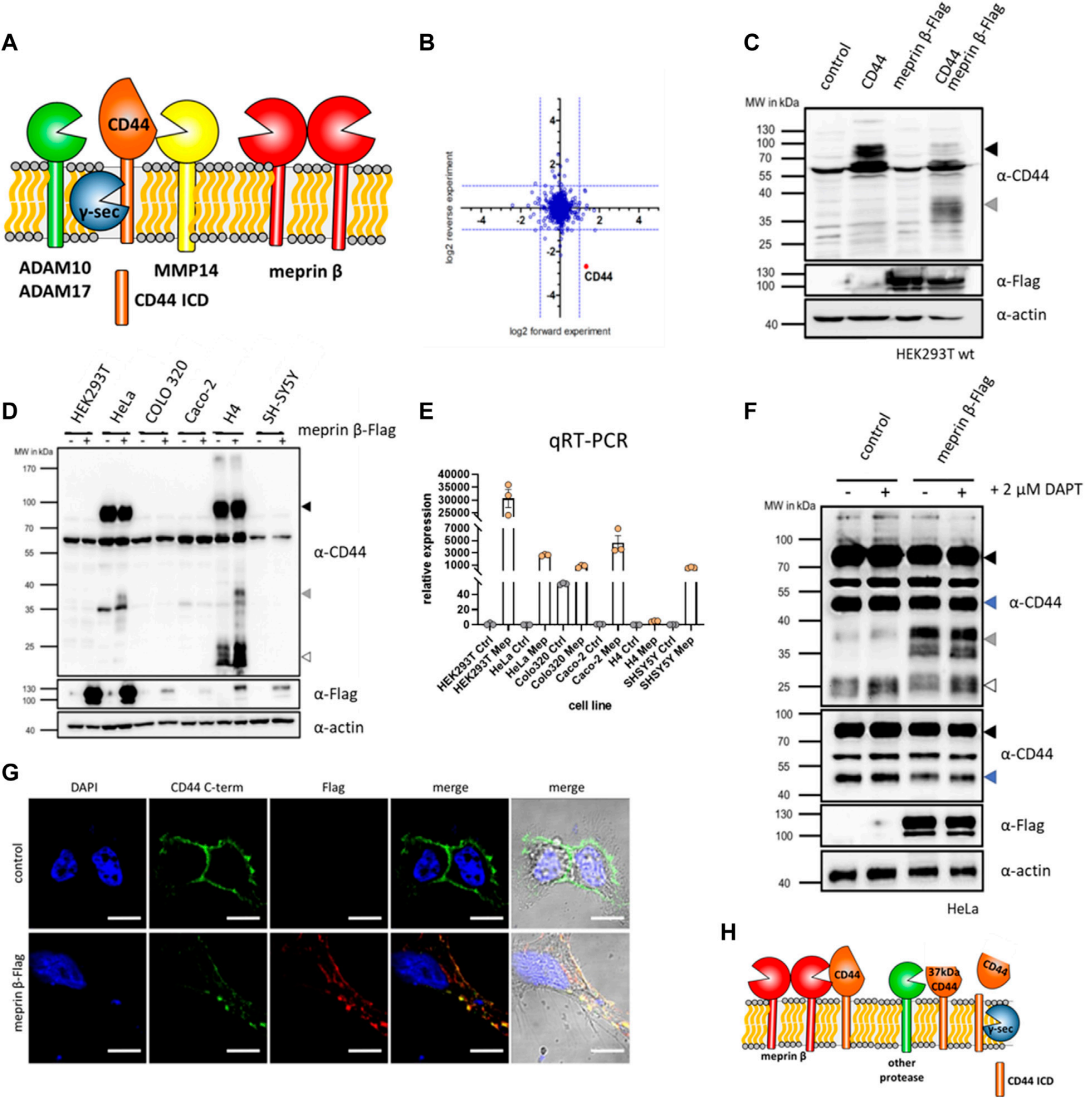
CD44 cleavage by meprin β. **(A)** Cartoon of different proteases cleaving CD44 at the cell surface. **(B)** Quantitative mass spectrometry shows reduced levels of CD44 on fibroblasts stimulated with soluble meprin β. **(C)** Western blot of HEK93T cell lysates shows a 37 kDa truncated version of CD44 upon dual expression of CD44 and meprin β. **(D)** Western blot of different cell lines with or without transfection of meprin β blotted for endogenous CD44 (black arrow = full-length CD44, grey arrow = meprin β dependent cleavage fragment, white arrow = additional CTFs of CD44). **(E)** Expression of meprin β in different cell lines. **(F)** Control HeLa cells or transfected with meprin β and additionally treated with the γ-secretase inhibitor DAPT shows the accumulation of a 25 kDa CTF (white arrow) of CD44 and a meprin β dependent decrease in a 50 kDa (blue arrow) CD44 fragment. **(G)** Immunofluorescence of control HeLa cells or meprin β expressing ones stained against endogenous CD44 (green), meprin β (red) and the nucleus (blue). **(H)** Summarizing cartoon of meprin β dependent CD44 cleavage that generates a ∼37 kDa large trimmed CD44 at the cell surface. This fragment might be processed by a different protease to generate a 25 kDa γ-secretase dependent CTF. However, generation of the γ-secretase dependent CTF seems independent of meprin β.

Cluster of differentiation 44 (CD44) is ubiquitously expressed and the main cell surface receptor for hyaluronan in the human body (Aruffo et al., 1990). CD44 is a highly glycosylated type 1 transmembrane protein and it is implicated in many different cellular processes such as cell-cell and cell-matrix contacts, cell motility, cell signaling and can also act as a coreceptor for growth factors and cytokines (Ponta et al., 2003; Orian-Rousseau et al., 2002). Among these broad implications in cellular processes, CD44 represents also a common cancer stem cell marker and is highly expressed on many different cancer cells (Thapa and Wilson, 2016; Jaggupilli and Elkord, 2012; Wang et al., 2019). For several cell lines including osteosarcoma cells high levels of CD44 correlate with poor prognosis and metastasis and deletion/knockout (KO) of CD44 resulted in reduced tumor cell invasion and proliferation (Liu et al., 2018; Xiao et al., 2018). CD44 is encoded by a highly conserved gene located on chromosome 11 in humans or chromosome two in mice, respectively (Goldstein et al., 1989; Gao et al., 1997; Chen et al., 2018). The standard form of CD44 is encoded by 10 constant exons (exons 1–5, 16–20), whereas additional variable exons v1-v10 are inserted between exon five and exon 16 and then encode for variable isoforms of CD44 (CD44v). Exon v1 is only expressed in mice but not in humans, resulting in 19 exons in humans and 20 exons in mice and variants of CD44 ranging from molecular weights of 85–200 kDa due to alternative splicing and glycosylation (Brown et al., 1991; Screaton et al., 1992; Screaton et al., 1993; Rall and Rustgi, 1995; Naor et al., 2002). CD44-mediated processes like cell-matrix adhesion can be further influenced by posttranslational modifications such as N- and O-linked glycosylation or glycosaminoglycanation by the addition of heparan sulfate or chondroitin sulfate (Bennett et al., 1995; Bartolazzi et al., 1996). Besides hyaluronan, CD44 binds to other components of the extracellular matrix (ECM) such as osteopontin (Weber et al., 1996), collagens (Knutson et al., 1996), fibronectin (Iczkowski et al., 2006) and several soluble matrix metalloproteinases (MMPs) such as MMP2, MMP7 and MMP9 (Bourguignon et al., 1998; Takahashi et al., 1999; Yu et al., 2002). In addition to soluble MMPs, CD44 can form a protein complex with the membrane bound MMP14 (aka MT1-MMP) at the cell surface, thereby directing it to the leading edge of migrating cells and promoting tumor cell invasion (Mori et al., 2002). Multiple stimuli result in cleavage of CD44 at the cell surface at different sites and in the release of extracellular fragments. Membrane proximal cleavage of CD44 (shedding) is followed by the generation of an intracellular domain (ICD) through intramembrane cleavage by the presenilin/γ-secretase complex capable of changing target gene expression *via* nuclear translocation (Okamoto et al., 2001; Murakami et al., 2003). Among several other target genes, expression of MMP2 and MMP9 can be influenced by the CD44 ICD (Zhang et al., 2002; Miletti-Gonzalez et al., 2012). For elevated serum levels of the soluble extracellular domain of CD44v6 and the CD44 ICD an association with tumor burden, metastasis and poor prognosis in several cancers including gastric, colorectal or breast cancer have been shown (Guo et al., 1994; Sheen-Chen et al., 1999; Yamane et al., 1999; Stamenkovic and Yu, 2009). Several proteinases implicated in proteolytic processing of CD44 are already identified including A Disintegrin And Metalloproteinase 10 (ADAM10) and ADAM17 as well as MMP14 (Kajita et al., 2001; Ueda et al., 2003; Nagano et al., 2004; Nagano and Saya, 2004; Nakamura et al., 2004) (Figure 1A). It is still unclear which proteinase represents the major sheddase of CD44 and how these proteinases interact with each other. It was shown that ADAM10 or ADAM17-mediated cleavage of CD44 is triggered upon different stimulation e.g., by Ca^2+^ influx or protein kinase C (PKC) activation (Nagano et al., 2004), whereas a study on melanoma cells indicated that constitutive and endogenous shedding of CD44 is mediated by ADAM10 (Anderegg et al., 2009). Studies utilizing fibrosarcoma and gastric carcinoma cell lines with downregulated expression of MMP14 showed impaired tumor cell migration and invasion (Ueda et al., 2003). During cancer progression and metastasis, epithelial to mesenchymal transition (EMT) is controlled by CD44 undergoing isoform switching (Brown et al., 2011). This process is influenced by different proteins including the epithelial splicing regulatory protein 1 (ESRP1) and the transcription factor Snail family transcriptional repressor 1 (SNAI1) (Warzecha et al., 2009; Reinke et al., 2012; Yae et al., 2012; Chen et al., 2017). SNAI1 is a downstream target of CD44 and capable of repressing the transcription of E-cadherin and ESRP1, thereby influencing EMT. SNAI1 has been shown to be a downstream target of MMP14 gene expression (Reinke et al., 2012; Jiang et al., 2015; Chen et al., 2017). In turn, decreased levels of ESRP1 correlate with an isoform switch towards CD44s, required for EMT and metastasis (Yae et al., 2012).

In this study we identify and characterize CD44 cleavage by the metalloproteinase meprin β and found a ∼37 kDa C-terminal fragment as the main meprin β dependent cleavage product remaining at the cell surface. Meprin β is a membrane bound metalloproteinase that is primarily expressed in the gut, kidney and on different immune cells (Arnold et al., 2017a; Peters et al., 2019; Li et al., 2022). Single amino acid exchange variants of meprin β that were identified in different tumor entities, change the cell surface activity and localization of meprin β and thereby influence substrate cleavage (Schaffler et al., 2019; Gellrich et al., 2021). In the past we identified other cell surface proteins/receptors such as the interleukin-6 receptor (IL-6R) (Arnold et al., 2017b), CD99 (Bedau et al., 2017a; Bedau et al., 2017b), Triggering receptor expressed on myeloid cells 2 (TREM2) (Berner et al., 2020; Schwarz et al., 2022) and CD109 (Luckstadt et al., 2021) as proteolytic targets of membrane bound meprin β. In addition to meprin β, we also evaluate the known sheddases ADAM10, ADAM17 and MMP14 for endogenous CD44 cleavage. Applying state-of-the-art CRISPR/Cas9 technology and analyzing transcriptional levels of different MMPs in cells and tissues (GTEx data), we identify MMP14, MMP2, MMP9 and MMP25 as transcriptional targets of the ADAM10/CD44 axis. In functional cell experiments, we show that cells deficient for CD44 or MMP14 show similar behavior in cell adhesion, proliferation, migration and spheroid formation. Thus suggesting a functional complex between CD44 and MMP14 at the cell surface that could then form the basis for the assembly of a large proteolytic hub.

## 2 Results

### 2.1 Meprin β cleaves CD44 at the cell surface

As a cell surface receptor, CD44 can be shed by different proteases. We identified CD44 as a new putative proteolytic substrate of meprin β, when we treated fibroblasts with active soluble meprin β and analyzed globally downregulated proteins by mass spectrometry (Luckstadt et al., 2021) (Figure 1B). To confirm CD44 as a substrate for membrane bound meprin β, we utilized HEK293T cells and expressed CD44 and meprin β individually or together. Western blot analysis of cell lysates revealed an additional membrane tethered C-terminal cleavage fragment (CTF) of CD44 around 37 kDa only present in samples derived from cells that expressed both, CD44 and meprin β (Figure 1C). Thus, soluble and membrane bound meprin β cleave CD44 at the cell surface. To further elaborate on this cleavage, we analyzed different cell lines for endogenous expression of CD44 to omit transient expression of more than one protein at a time and work with endogenous substrate levels. We identified HeLa (cervix carcinoma) and H4 (neuroglioma) cells to express CD44 at detectable levels in Western blot analysis and could also confirm the specific cleavage fragment of CD44 at 37 kDa after transient expression of meprin β in these two cell lines (Figure 1D). To analyze the endogenous expression of meprin β in these cells, we then performed quantitative real-time PCR (qRT-PCR) and found almost no endogenous expression for this protease in HeLa or H4 cells that both express CD44 (Figure 1E). As HeLa cells showed a better expression of meprin β after transient transfection than H4 cells, we continued our subsequent experiments with HeLa cells. Moonlighting (translocation into the nucleus) of the CTF of CD44 is one major function that regulates the expression of different target genes (Zhang et al., 2002; Miletti-Gonzalez et al., 2012). Prerequisite for the release of an ICD that can then translocate to the nucleus, is cleavage of the previously generated CTF by the presenilin/γ-secretase complex (Okamoto et al., 2001; Murakami et al., 2003). To analyze the capability of meprin β to generate such a γ-secretase dependent CTF, we transfected HeLa cells with meprin β and then blocked γ-secretase cleavage with DAPT (Yang et al., 2008). Both, transfected and non-transfected cells showed an accumulation of CTFs at around 25 kDa, but no increase in CTFs was detected in meprin β expressing cells (Figure 1F). Additionally, we determined transcriptional levels of CD44 and the two known sheddases of CD44, namely ADAM10 and MMP14. We did not detect a significant increase for any of them (Supplementary Figure S1). Thus, the ultimate generation of a γ-secretase dependent CD44 CTF seems to be independent of meprin β in HeLa cells. A second CD44 cleavage fragment that runs at ∼50 kDa seems to also serve as a proteolytic substrate of meprin β, as we detect decreasing levels in cells additionally expressing meprin β (Figure 1F). To analyze the cellular localization of CD44 and meprin β, immunofluorescence staining of both proteins was conducted in HeLa cells transfected with meprin β. Both proteins expressed at the cell surface and co-localized, indicating possible direct interaction (Figure 1G). Thus, we can summarize that meprin β cleaves CD44 at the cell surface of HeLa cells and generates a cell surface bound ∼37 kDa CD44 species. This ∼37 kDa CD44 is likely not targeted by the presenilin/γ-secretase complex and generation of the ICD upon γ-secretase cleavage seems independent of previous cleavage by meprin β in HeLa cells (Figure 1H). The cleavage pattern seems different in H4 cells (Figure 1D), however, both cell lines require transient expression of meprin β as HeLa as well as H4 cells have a very low endogenous expression of meprin β (Figure 1E). Thus, both cell lines might not represent the ideal model to evaluate the endogenous influence of meprin β on CD44.

### 2.2 CRISPR/Cas9 reveals a complex transcriptional network for CD44 shedding proteases

**FIGURE 2:**
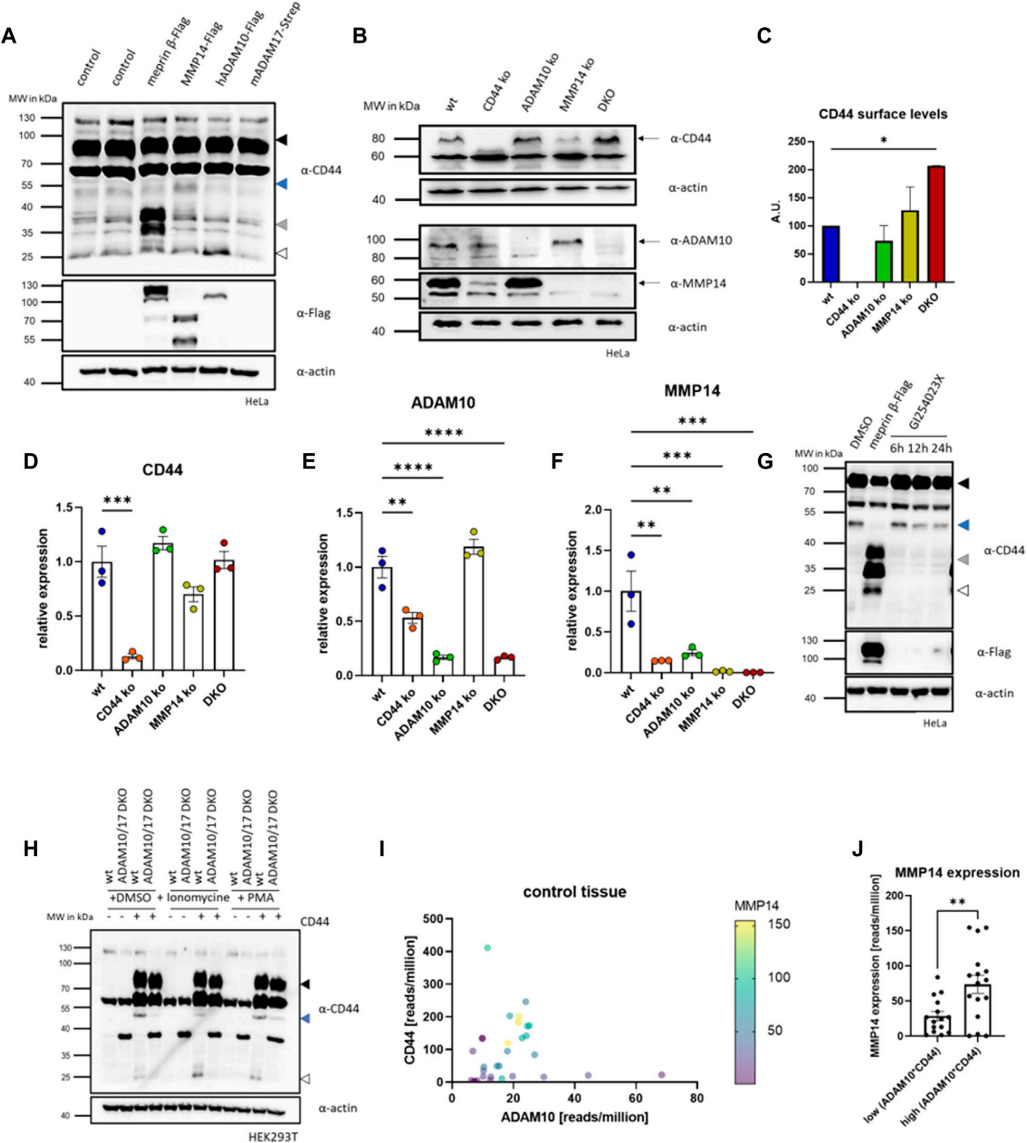
CRISPR/Cas9 of CD44 and shedding enzymes. **(A)** Western blot of HeLa cells expressing different CD44 cleaving proteases. Arrows indicate different membrane associated CD44 cleavage fragments (black = full-length CD44, blue = 50 kDa CD44, grey = 37 kDa fragment, white = 25 kDa CTF). **(B)** Western blot of different CRISPR/Cas9 generated HeLa cell lines deficient for CD44 (CD44 ko), ADAM10 (ADAM10 ko), MMMP14 (MMP14 ko) and ADAM10 and MMP14 (DKO). **(C)** FACS analysis of CD44 cell surface levels (n = 3, values with SEM, one-way ANOVA). **(D)** Transcriptional level of CD44 determined by qRT-PCR in the different CRISPR/Cas9 generated cell lines (n = 3, values with SEM, one-way ANOVA). **(E)** Transcriptional level of ADAM10 determined by qRT-PCR in the different CRISPR/Cas9 generated cell lines (n = 3, values with SEM, one-way ANOVA). **(F)** Transcriptional level of MMP14 determined by qRT-PCR in the different CRISPR/Cas9 generated cell lines (n = 3, values with SEM, one-way ANOVA). **(G)** Western blot of HeLa cell lysates against CD44 in cells expressing meprin β or incubated with the ADAM10 inhibitor GI254023X for different time periods (arrows as in a). **(H)** Western blot of HEK293T wt and ADAM10 and ADAM17 double deficient (DKO ADAM10/17) HEK293T cells against CD44. Cells were transfected with CD44 and additionally cleavage of CD44 was induced by stimulation of endogenous ADAM10 (Ionomycine) or ADAM17 (PMA). **(I)** Analysis of GTEx (gene tissue expression) data derived from the Gepia2 server. Expression values are plotted against CD44 (*y*-axis) and ADAM10 (*x*-axis). Expression levels of MMP14 are shown from purple (low expression) to yellow (high expression). **(J)** GTEx data for CD44 and ADAM10 was multiplied and samples were sorted into upper and lower median. MMP14 expression in the upper median is significantly higher than in the lower median (n = 15 (lower median) and n = 16 (upper median), values shown as mean with SEM, unpaired Student’s t-test). *p < 0.05, **p < 0.01, ***p < 0.001 ****p < 0.0001.

From our cleavage experiments with meprin β, we concluded that other endogenous proteases cleave CD44 continuously. This is not surprising, as other proteases were described to process CD44 such as ADAM10 and ADAM17 and MMP14 (Kajita et al., 2001; Ueda et al., 2003; Nagano and Saya, 2004; Nakamura et al., 2004). To verify this in our HeLa cell system, we expressed meprin β, MMP14, ADAM10 and ADAM17 transiently and analyzed the resulting cleavage fragments (Figure 2A). For meprin β we identified fragments described above for HEK293T cells at ∼37 kDa, for MMP14 we identified a 50 kDa fragment as the major cleavage product and for ADAM10 a 25 kDa C-terminal fragment appears to be the major cleavage product (Figure 2A). For ADAM17 we did not detect an additional cleavage fragment (Figure 2A). To analyze individual effects of CD44, MMP14 and ADAM10 in HeLa cells, we then decided to produce respective knockout cell lines by CRISPR/Cas9 (Supplementary Figure S2A). Additionally, we also produced a HeLa cell line lacking MMP14 and ADAM10, which we will term DKO (double knock out) in the following (Supplementary Figure S2A). We assessed the expression levels of all our CRISPR/Cas9 targets (CD44, MMP14 and ADAM10) in five different cell lines (wild-type (wt), CD44 ko, MMP14 ko, ADAM10 ko and DKO) by western blot and could thus confirm a successful depletion of the respective proteins in all cell lines (Figure 2B). In DKO cells, missing the two CD44 sheddases ADAM10 and MMP14, CD44 protein levels appeared increased in western blot analysis (Figure 2B), which was confirmed by FACS experiments stained against cell surface CD44 (Figure 2C). We identified CD44 cell surface levels on DKO cells twice as high as on wt HeLa cells (Figure 2C). These experiments confirm MMP14 and ADAM10 as endogenous sheddases of CD44 at the cell surface. For ADAM10 the protein signal was reduced to about 50% in western blot analysis of CD44 ko cells, but unchanged in MMP14 ko cells (Figure 2B). For MMP14 reduced protein levels could be observed in CD44 ko cells, while MMP14 levels in ADAM10 ko cells compared to those in HeLa wt cells (Figure 2B). To additionally assess changes on the transcriptional level, qRT-PCR experiments were conducted. All genes targeted by CRISPR/Cas9 were strongly reduced in qRT-PCR analyses (Figures 2D–F). Expression levels of CD44 were detected as in wt HeLa cells in cell lines missing one or two of the proteases, thus deletion of one or both of the proteases does not influence CD44 expression (Figure 2D). For ADAM10, we found a 50% reduced expression compared to wt HeLa cells upon deletion of CD44 (Figure 2E). This reduction on the mRNA level fits nicely to the western blot analysis that also reported a reduced protein level for ADAM10 (Figure 2B). Biggest transcriptional effects were detected for the transcriptional levels of MMP14, as we measured significantly reduced levels in all ko cell lines generated (Figure 2F).

To test if the generation of the MMP14 dependent 50 kDa cleavage fragment of CD44 requires proteolytic activity of ADAM10, we treated HeLa cells with the ADAM10 inhibitor GI (GI254023X) in a time course experiment. Indeed, we found reduced levels of the MMP14 specific cleavage fragment after 12 and 24 h (Figure 2G). To further test the ADAM10 dependent induction of this cleavage fragment, we compared HEK293T wt cells and a HEK293T line deficient for ADAM10 and ADAM17 (Riethmueller et al., 2016). After expression of CD44 in both, a markedly stronger signal for the 50 kDa CD44 fragment appeared in HEK293T wt cells when compared to the ADAM10/17 deficient cell line (Figure 2H). To elaborate if the expression of MMP14 depends on CD44 and ADAM10 in different healthy tissues, we explored publicly available transcriptional data deposited in the GTEx database, which we accessed through Gepia2 (Tang et al., 2019). When plotting the transcriptional levels of MMP14 in dependence of ADAM10 and CD44 a clear correlation could be identified (Figure 2I). Separating tissues at median into those with high and low ADAM10*CD44 (calculated as product of reads/million for ADAM10 and CD44) expression showed significantly increased MMP14 expression levels for the upper 50% when compared to the lower 50% (Figure 2J). Thus, we conclude that MMP14 expression seems to depend upon CD44 and ADAM10 under physiological conditions in the tissues analyzed.

### 2.3 Different MMPs are transcriptionally regulated *via* CD44

**FIGURE 3:**
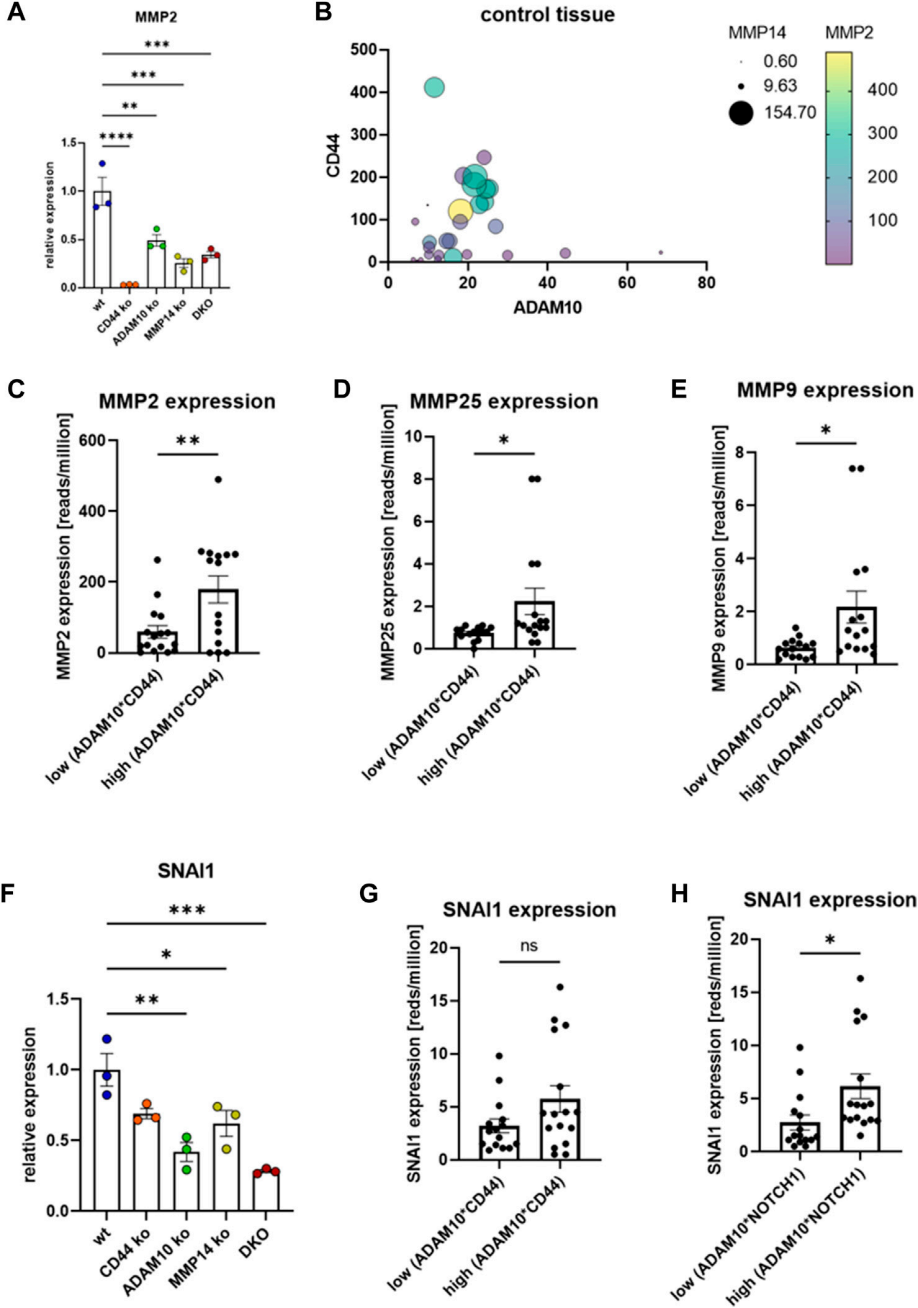
Transcriptional regulation of selected MMPs. **(A)** Transcriptional regulation of MMP2 in different CRISPR/Cas9 generated cell lines (n = 3, values are shown with SEM, one-way ANOVA). **(B)** Transcriptional regulation of MMP2 in dependency of CD44, ADAM10 and MMP14 analyzed in GTEx data. **(C)** Transcriptional regulation of MMP2 in dependency of ADAM10 and CD44 (n = 15 (lower median) and n = 16 (upper median), values shown as mean with SEM, unpaired Student’s t-test). **(D)** Transcriptional regulation of MMP25 in dependency of ADAM10 and CD44 (n = 15 (lower median) and n = 16 (upper median), values shown as mean with SEM, unpaired Student’s t-test). **(E)** Transcriptional regulation of MMP9 in dependency of ADAM10 and CD44 (n = 15 (lower median) and n = 16 (upper median), values shown as mean with SEM, unpaired Student’s t-test). **(F)** The expression of SNAI1 is significantly reduced in cells deficient for MMP14, ADAM10 or both proteases (n = 3, values are shown with SEM, one-way ANOVA). **(G)** The expression of SNAI1 does not depend on the expression of ADAM10/CD44 (n = 15 (lower median) and n = 16 (upper median), values shown as mean with SEM, unpaired Student’s t-test). **(H)** The expression of SNAI1 differs significantly when sorted by the expression level of ADAM10*NOTCH1 (n = 15 (lower median) and n = 16 (upper median), values shown as mean with SEM, unpaired Student’s t-test). *p < 0.05, **p < 0.01, ***p < 0.001 ****p < 0.0001.

For MMP2, one of the soluble MMPs, a transcriptional effect through CD44 cleavage was also suggested (Zhang et al., 2002; Zoller, 2011). We could confirm this finding in our CRISPR/Cas9 ko cell lines and measured almost no transcriptional activity for MMP2 in cells deficient for CD44 (Figure 3A). In the cell lines deficient for MMP14, ADAM10 or DKO a significant reduction for MMP2 expression was detected (Figure 3A). Blotting expressional data from the GTEx repository revealed the interdependency of MMP2 on the expression of CD44, ADAM10 and MMP14 (Figure 3B). Separating MMP2 expression at median into an upper and a lower ADAM10*CD44 group shows a significant difference between both groups and indicates MMP2 expression to be regulated by ADAM10 and CD44 (Figure 3C). In GTEx data we now blotted all MMPs and TIMPs (Tissue Inhibitors of MMPs) against the quotient of ADAM10*CD44 and identified MMP25 (Figure 3D) and MMP9 (Figure 3E) to show the same effect as shown for MMP2. MMP25 is membrane tethered and was shown to activate MMP2, while MMP9 is soluble and can be activated by MMP14 (Li et al., 2017). CD44 is known to not only influence MMP transcription, but also SNAI1 (SNAIL) (Yae et al., 2012). SNAI1 is an important transcriptional repressor for e.g., cadherins and plays an important role during EMT. In our HeLa CRISPR/Cas9 cell lines we found SNAI1 majorly downregulated in ADAM10 ko and DKO cells and to a smaller degree in MMP14 deficient cells (Figure 3F). In CD44 deficient cells no significant downregulation could be determined (Figure 3F), which is in line with GTEx data that shows no significant relation between SNAI1 and ADAM10*CD44 (Figure 3G). As strong effects were observed in cells deficient for ADAM10 and ADAM10 is the major sheddase for NOTCH1 to release the NICD (Notch intracellular domain) in a γ-secretase dependent manner (Tian et al., 2008; Gellrich et al., 2021), we tested for a correlation between SNAI1 and ADAM10*NOTCH1 expression. Indeed, we identified a significantly higher SNAI1 expression in GTEx data in the upper half sorted for ADAM10*NOTCH1 when compared to the lower half (Figure 3H). Taken together, we can show that CD44 shedding by ADAM10 is an important step to release the γ-secretase dependent CTF and induce expression of MMP14 and MMP2. GTEx data confirmed this regulatory effect and additionally suggests MMP9 and MMP25 as targets of the ADAM10/CD44 axis. Identification of this regulatory effect in GTEx data might furthermore suggests a common regulatory effect of this ADAM10/CD44 axis across many tissues under physiological conditions.

### 2.4 CRISPR/Cas9 ko cell lines reveal distinct functions for CD44 and its shedding enzymes

**FIGURE 4:**
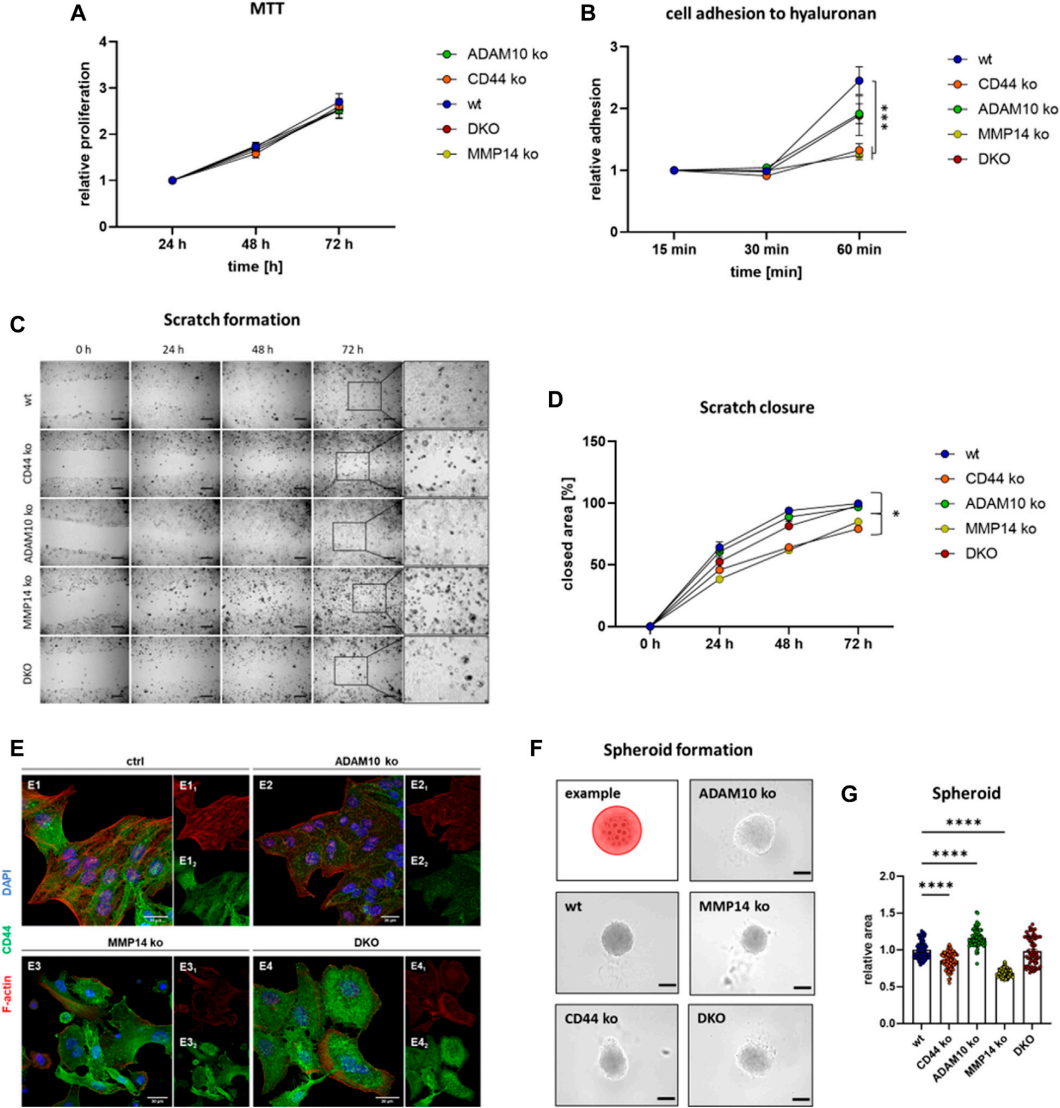
Functional consequences for loss of CD44 or its shedding enzymes. **(A)** Cell viability/proliferation determined with an MTT assay reveals no difference between the different cell lines (n = 15 for wt, CD44 ko, ADAM10 ko and MMP14 ko, n = 8 for DKO, values shown with SEM, two-way ANOVA). **(B)** Cell adhesion to hyaluronan as determined utilizing a plate and wash assay (n = 9, values shown with SEM, two-way ANOVA, complete statistical analysis in Supplementary Table S1). **(C)** Closure of a previously applied scratch was determined at 24, 48, and 72 h. Inserts (at right) show magnification of the previous scratch area at 72 h **(D)** Quantification of the scratch assay shown in **(C)** (n = 11, values shown with SEM, two-way ANOVA, complete statistical analysis in Supplementary Table S2). **(E)** Immunofluorescence microscopy against CD44 (green) and F-actin (Phalloidine, red) and the nucleus (blue). **(F)** Tumor spheroid formation assay with an exemplary cartoon showing how the spheroid area was determined (upper left corner). **(G)** Quantification of individual tumor spheroid areas (n = 62–77, individual values shown as mean with SEM, one-way ANOVA, ****p < 0.0001).

In order to compare different cell lines in terms of adhesion, proliferation, migration and spheroid formation, we tested cell viability/proliferation first (Figure 4A). For this, we performed a MTT-assay and found no differences between all cell lines at 24, 48, and 72 h (Figure 4A). Thus, functional differences should not be linked to differences in cell viability or proliferation. As a known receptor for hyaluronan, CD44 is an important adhesion molecule (Aruffo et al., 1990). To assess differences in cellular attachment to this matrix protein, we coated cell culture dishes with hyaluronan and performed a plate and wash assay. Cell attachment was measured after 15, 30, and 60 min and we observed significantly lower attachment for cells deficient for CD44 and MMP14 when compared to the other cell lines (Figure 4B). We then measured the ability of the different cell lines to cover a scratch introduced into a confluent monolayer and measured the remaining area after 24, 48, and 72 h (Figure 4C). While ADAM10 ko and DKO cells performed as the parental HeLa wt cells, CD44 ko and MMP14 ko cell lines required more time to cover the same area (Figure 4D). However, there is no difference between CD44 ko and MMP14 ko cells (Figure 4D). In higher magnification, we saw that CD44 and MMP14 deficient cells did not form a homogenous migration front in our scratch assay experiments, but rather migrated as single cells (Figure 4C, magnification at 72 h). Thus, we stained all above-mentioned cell lines for endogenous CD44, using a specific primary antibody, and also for F-actin, to detect cell morphology, as well as for DNA (nucleus) using DAPI (Figure 4E). Control HeLa wt cells grew as sheets, with tight cell-cell contacts, and showed mostly intracellular CD44 staining. ADAM10 ko cells exhibited a similar phenotype, although with less intense CD44 signal. In contrast, MMP14 ko cells featured a more elongated morphology, often disrupted cell-cell adhesion, and pronounced plasma-membrane-associated CD44 signals. DKO cells showed an intermediate phenotype, with discontinuous cell-cell adhesion. In addition, CD44 was not uniformly distributed in the cytoplasm, and cells were often bigger than their control or single ko counterparts.

To assess cell-cell contact formation further, we utilized a 3D cell culture system and analyzed the ability of our CRISPR/Cas9 generated cell lines to form tumor spheroids (Figure 4F). For this, equal amounts of cells were seeded into low-attachment plates and spheroid formation was determined after 96 h. To compare the different spheroids, images were taken and the area of spheroids was measured. Spheroids derived from DKO cells showed no significant difference to those derived from control HeLa wt cells (Figure 4G). ADAM10 deficient cells formed significantly larger spheroids, while CD44 ko and MMP14 ko cells assembled significantly smaller ones, when compared to those from HeLa wt cells (Figure 4G). In summary, cells deficient for CD44 show decreased abilities in cell attachment, migration and spheroid formation. Most of these effects can also be seen for MMP14 deficient cells. However, DKO cells lacking ADAM10 and MMP14 seem to perform differently. One explanation could be the compensatory upregulation of other shedding enzymes. To test this hypothesis, we performed qRT-PCR experiments in all cell lines and assessed the expression level of ADAM17 and meprin β. While ADAM17 levels remain stable in all cell lines, meprin β expression is induced in MMP14 ko and even more in DKO cells (Supplementary Figures S2B, C).

## 3 Discussion

**FIGURE 5:**
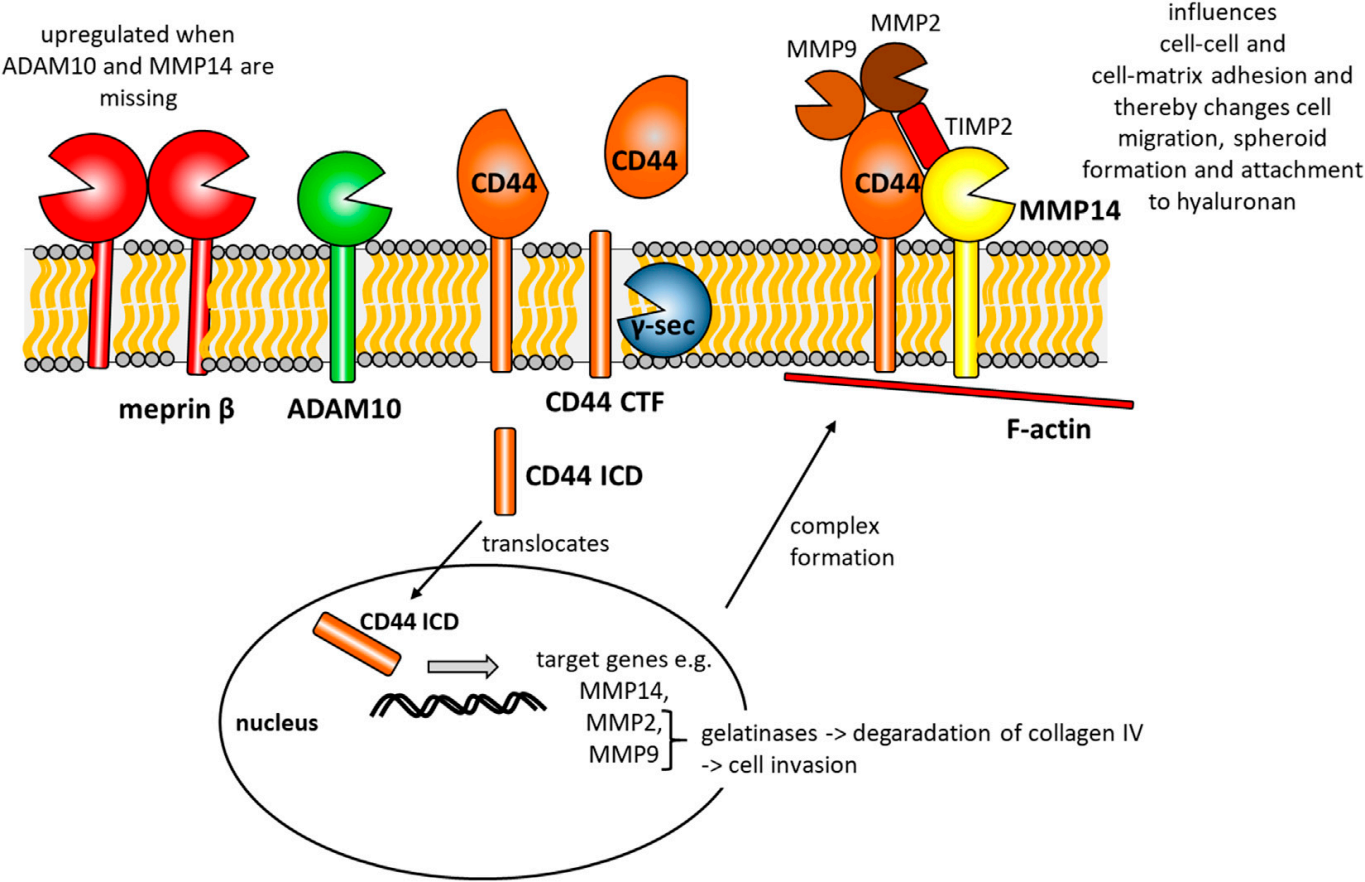
Summarizing cartoon of our findings combined with data published by other groups before. CD44 is cleaved by ADAM10 to generate a CD44 C-terminal fragment (CD44 CTF). This CTF is then prone to y-secretase (y-sec) cleavage within the membrane, which generates a CD44 intracellular domain (CD44 ICD). This ICD translocates to the nucleus and induces the expression of e.g. MMP14, MMP2 and MMP9. At the cell surface CD44, MMP14, MMP2, MMP9 and the tissue inhibitor of metalloproteinases 2 (TIMP2) form a multi-protein complex and attach to intracellular F-actin. This complex influences cell-cell and cell-matrix contacts and thereby changes cell migration, spheroid formation and attachment to hyaluronan.

In this manuscript, we describe the metalloproteinase meprin β as a new sheddase for CD44. Meprin β cleaves CD44 in its soluble and in its membrane bound form, which is a major difference to the IL-6R that is cleaved by membrane bound but not soluble meprin β (Arnold et al., 2017b). We found that cleavage by meprin β generates CTFs of different size with the main fragment ranging at around ∼37 kDa. However, the generation of a γ-secretase dependent CTF around 25 kDa seems to be independent of meprin β at least in HeLa cells. Of note, the MMP14 dependent CD44 cleavage fragment around 50 kDa is reduced in meprin β expressing cells. Cleavage of substrates, as seen here for CD44, at positions distant to the transmembrane region is common for meprin β and has been shown for CD99 (Bedau et al., 2017a; Bedau et al., 2017b), TREM2 (Berner et al., 2020) and CD109 (Luckstadt et al., 2021) before. This is a major difference to ADAM10, which cleaves within close proximity to the membrane (Kato et al., 2018). Although, we describe meprin β as a new sheddase for CD44, we are missing information on the endogenous interaction between meprin β and CD44. This matter can only be solved in a cell line that expresses both proteins at sufficient endogenous level to trigger shedding of CD44 by meprin β. At this point we are missing such a cell line. Our experiments in HeLa cells indicate ADAM10 as the important endogenous sheddase for CD44 cleavage to induce processing by the γ-secretase complex and generation of an ICD. This mode of action is similar to NOTCH cleavage by ADAM10 where a γ-secretase dependent CTF is further processed to an ICD that then controls target gene expression (Pan and Rubin, 1997; Medina and Dotti, 2003; Tian et al., 2008). Our data demonstrates a direct dependency of ADAM10 and CD44 on MMP14 and MMP2 expression in HeLa cells, which is in line with results deduced by analyzing GTEx data from various tissue origin. In GTEx data we also identified MMP9 as a possible target. Thus, we suggest shedding of CD44 by ADAM10 to control MMP14 and MMP2 expression as a general mechanism across different cell types and tissues (Figure 5). MMP2 and MMP9 are the two known gelatinases (Cabral-Pacheco et al., 2020) and their substrate repertoire includes the important basal membrane component collagen IV and the linker collagen VII between the basal membrane and the underlying ECM. It has been shown before that MMP14 and CD44 form a protein complex at the cell surface (Kajita et al., 2001; Mori et al., 2002), moreover a 50 kDa CD44 fragment could be co-precipitated with MMP14 in immunoprecipitation experiments (Mori et al., 2002). Interestingly, a similar fragment was proteolytically generated by MMP14 as we could show herein. On the mRNA and protein level, we find strongly reduced levels of MMP14 in CD44 ko cells, which might explain synonymous outcome in functional assays of both cell lines. CD44 and MMP14 deficient cells show inferior ability to attach to hyaluronan, re-populate a scratched of area in a scratch assay experiment and show inferior growth in spheroid formation experiments. However, cell viability and proliferation was not different and would not explain the observed differences. As CD44 is the primary receptor for hyaluronan (Aruffo et al., 1990), inferior ability to attach to it was somehow expected from our western blot results. To our surprise, we found that effects detected in MMP14 deficient cells are lost in ADAM10/MMP14 double deficient cells. This could be explained by an effect that we observed for the transcriptional regulation of meprin β. In these double deficient cells meprin β is upregulated. This compensatory effect in proteolytic networks has been described before and other proteases can take over substrate cleavage in cases where the primarily cleaving protease is missing (Li et al., 2022). Another effect that is often seen in condition where a sheddase is missing, is the release of proteolytic substrates on extracellular vesicles. This was shown for the ADAM10 substrate prion protein (Linsenmeier et al., 2018), the ADAM10/ADAM17 and meprin β substrate IL-6R (Arnold et al., 2020) and the meprin β substrate CD109 (Luckstadt et al., 2021). Whether this effect holds true for CD44 and has any biological effect on cells, has to be elucidated in future research projects. CD44 expression in tumor cells is often associated with increased invasiveness in surrounding tissue (Hou et al., 2019; Wang et al., 2019) and different approaches were used to block CD44 (Montgomery et al., 2012; Gao et al., 2015) resulting in a less invasive phenotype. The increased invasiveness is most likely due to the assembly of a proteolytic complex of CD44, MMP14, MMP2, MMP9 and other proteases. Especially the two gelatinases MMP2 and MMP9 with their potency in cleaving collagen IV, might increase the potential of cells to invade through the basal membrane. As we identified ADAM10 as a key protease to induce CD44 dependent transcriptional upregulation of MMP14 and MMP2, inhibition of CD44 cleavage by ADAM10 might be a future translational approach to prevent tumor cell migration and invasion.

In summary, we identify meprin β as a new potent sheddase of CD44. In cell culture and knockout experiments we recognize ADAM10, but not ADAM17 as the most important endogenous sheddase for the generation of a γ-secretase dependent CTF in HeLa cells. The generation of this CTF is important for the transcriptional regulation of MMP14, MMP2 and MMP9 as our data and GTEx data indicates. A CD44 dependent assembly and expression of a potent proteolytic complex at the cell surface could explain the pro-invasive effects associated with an increased CD44 expression in tumor cells. Furthermore, the identification of the ADAM10 dependent generation of CD44 CTFs, might open new routes in translational approaches that could block CD44 cleavage by ADAM10 or other proteases.

## 4 Materials and methods

Basic chemicals were purchased from Carl Roth GmbH & Co. KG, Karlsruhe, Germany and disposable material were obtained from SARSTEDT AG & Co. KG, Nümbrecht, Germany, unless otherwise stated.

### 4.1 Cell lines, cell culture and transient transfection

HEK293T, HeLa, COLO320, Caco-2 and H4 cells were cultivated in Dulbecco’s Modified Eagle’s Medium high-glucose medium (DMEM, Gibco™, Carlsbad, CA, United States) containing 10% fetal bovine serum (FBS, PANBiotech, Aidenbach, Germany) and 1% Penicillin/Streptomycin (Applied Biological Materials Inc., Richmond, Canada). SH-SY5Y cells were cultivated in Dulbecco’s Modified Eagle Medium: Nutrient Mixture F-12 (DMEM/F-12, Gibco™, Carlsbad, CA, United States) containing 10% fetal bovine serum (FBS, PANBiotech, Aidenbach, Germany) and 1% Penicillin/Streptomycin (Applied Biological Materials Inc., Richmond, Canada). All cells were cultured under constant conditions at 37°C, with 5% CO_2_ and 95% relative humidity. Transient transfection was performed after a 24 h incubation period of cells seeded at a concentration of 1 × 10^6^ cells in a 10 cm cell culture dish. For each 10 cm cell culture dish 500 µL DMEM medium without any supplements, 5 μg of plasmid DNA and 15 μL PEI MAX (1 mg/mL) were incubated for 30 min at room temperature in a 1.5 mL reaction tube and afterwards added to the cells and diluted 1:10 in cell culture medium. After 5 h of incubation at 37°C, with 5% CO_2_ and 95% relative humidity, fresh cell culture medium was added to the cells to obtain a 10 mL total volume. 24 h after transfection further experimental steps were taken. For other experiments, reagents were adjusted to smaller volumes. H4 and SH-SY5Y cells were transiently transfected using Lipofectamine™ 2000 (Thermo Fisher Scientific Inc., Waltham, MA, United States) or Effectene^®^ Transfection Reagent (Qiagen, Hilden, Germany) respectively, according to the manufacturer’s instructions. Ionomycine was used at 1 µM and PMA at 200 nM.

### 4.2 Proteomics

CD44 was identified in a proteomics screen as described before (Luckstadt et al., 2021). In brief, mouse kidney fibroblasts were incubated with soluble active meprin β or PBS as a control. Thereafter, cell membrane fractions were enriched and subjected to quantitative proteomics analysis. We identified CD109 (Luckstadt et al., 2021) and CD44 as downregulated on a global basis. Data available at the ProteomeXchange Consortium (http://proteomecentral.proteomexchange.org) *via* the PRIDE partner repository (Perez-Riverol et al., 2019) with the dataset identifier PXD023727.

### 4.3 Generation of CRISPR/Cas9 mediated knockout cells

Material for CRISPR/Cas9 mediated knockout of CD44, ADAM10 and MMP14 in HeLa cells were purchased from Synthego (Gene Knockout Kit v2, Synthego Corporation, Menlo Park, CA, United States). HeLa cells were transfected by electroporation using the Neon™ Transfection System (Thermo Fisher Scientific Inc., Waltham, MA, United States). Transfection was performed according the manufacturer’s instructions. Transfected cells were incubated in a 10 cm cell culture dish with 10 mL of cell culture medium without antibiotics. After 24 h antibiotics were added to the cell culture medium. To select the best knockout clone a single cell selection was performed. DNA was isolated using DNeasy Blood and Tissue Kit (Qiagen, Hilden, Germany) according to the manufacturer’s instructions and protein lysates were prepared for western bot experiments from each clone to further analyze the knockout efficiency. For HeLa cells lacking ADAM10 and MMP14 (DKO), the best single cell clone from HeLa cells lacking ADAM10 was used for transfection with mgRNA against MMP14. PCR’s were performed using 100 ng DNA, Q5 Hot Start High-Fidelity 2X Master Mix (NEB, Ipswich, MA, United States) and the primers listed in Table 1. PCR products were isolated from the agarose gel after electrophoretic separation by using a GeneJET Gel Extraction Kit (Thermo Fisher Scientific Inc., Waltham, MA, United States) according to manufacturer’s instructions. The purified PCR products were used for T7 endonuclease assay (NEB, Ipswich, MA, United States) or used for sequencing analysis by sanger sequencing by GATC services (Eurofins Genomics GmbH, Ebersberg, Germany). Sequencing results were analyzed by using the ICE CRISPR Analysis Tool (Synthego Corporation, Menlo Park, CA, United States).

**TABLE 1:** List of primers used for CRISPR/Cas9 and qRT-PCR experiments.

Target	Primer fwd 5′→ 3′	Primer rev 5′→ 3′
Cd44 (exon 2)	TGT​TGC​AGA​GCA​ATC​AAT​GGA​G	CCC​GCT​CTA​TGA​TTC​ACA​AAG​G
Cd44 (exon 2 nested)	AGC​TCC​TGA​CCT​CAG​GTG​AT	CCC​GCT​CTA​TGA​TTC​ACA​AAG​G
Adam10 (exon 2)	GCGGTTGGAATTACCCTC	GCTGGCACCAGTAGATAG
Mmp14 (exon 4)	AGC​CTG​AGG​ATC​CCT​TGT​TC	GAA​AGC​CAG​TCA​GTG​GGT​GA
Cd44 seq	ATTGTAGGCATGAGCCAC	
Adam10 seq	ATAGTGCTGGGATTAGGG	
Mmp14 seq	AGGGAAGGAGAATGTTGC	
hTub1a	TGG​CGT​TTT​GGA​AAG​ATA​CC	GGCATTGCCAATCTGGAC
hCd44v6	GCA​GTC​AAC​AGT​CGA​AGA​AGG	TGT​CCT​CCA​CAG​CTC​CAT​T
hAdam10	ATA​TTA​CGG​AAC​ACG​AGA​AGC​TG	TCA​ATC​GCT​TTA​ACA​TGA​CTG​G
hAdam17	CCT​TTC​TGC​GAG​AGG​GAA​C	CAC​CTT​GCA​GGA​GTT​GTC​AGT
hMmp14	CTG​TCA​GGA​ATG​AGG​ATC​TGA​A	AGG​GGT​CAC​TGG​AAT​GCT​C
hMep1b	TGA​CTC​TGA​TCT​CCT​AAA​GTT​GAA​TC	TGC​ACG​AGT​CCA​TAA​AAC​TCA
hMmp2	CCC​CAA​AAC​GGA​CAA​AGA​G	CTT​CAG​CAC​AAA​CAG​GTT​GC
hSnai1	TAC​AGC​GAG​CTG​CAG​GAC​T	ATCTCCGGAGGTGGGATG
hEsrp1	CCC​AAA​GAA​TGG​GTT​TGT​ATT​T	TGG​AGG​TTT​CAA​GAT​CAC​CAT

### 4.4 qRT-PCR

For qRT-PCR experiments 5 × 10^5^ HeLa cells were seeded into 6-Well cell culture plates. After 24 h mRNA was isolated using a NucleoSpin®RNA isolation kit (Macherey-Nagel GmbH & Co. KG, Düren, Germany) according to the manufacturer’s instructions. Afterwards mRNA was transcribed to cDNA using RevertAid^TM^Reverse Transcriptase (Thermo Fisher Scientific Inc., Waltham, MA, United States). Equal amounts (max. 1 μg) of mRNA were used, together with 2 µL oligo (dT)18-Primer (50 µM), 4 µL reaction buffer (5X), 0.5 µL RiboLock^TM^RNase Inhibitor (40 U/µL), 2 µL dNTPs (10 mM each) and 1 µL RevertAid^TM^Reverse Transcriptase (200 U/µL) (Thermo Fisher Scientific Inc., Waltham, MA, United States) and volume was adjusted to 20 µL with DEPC-treated water. After an incubation period of 1 h at 42°C, reaction was terminated by heating to 70°C for 10 min. Samples were prepared using PowerUp™ SYBR™ Green Mastermix (Thermo Fisher Scientific Inc., Waltham, MA, United States) and primers listed in Table 1. Samples were analyzed using an Applied Biosystems Prism 7,500 Real-Time PCR System.

### 4.5 Cell lysis, SDS-PAGE and western blot

Cells were seeded at a concentration of 1 × 10^6^ cells per 10 cm cell culture dish for western blot experiments. Stimulants and inhibitors were used and transient transfection was performed as described above. After 24 h of incubation cells were washed three times with PBS at 4°C and centrifuged at 1,200 g, resuspended in lysis buffer, containing EDTA, and incubated for 30 min on ice. To remove cell debris lysates were centrifuged for 15 min at 18,000 g at 4°C. Protein concentration of lysates was determined using Pierce™ BCA Protein Assay Kit (Thermo Fisher Scientific Inc., Waltham, MA, United States). Separation of proteins, including a prestained protein marker (Thermo Fisher Scientific Inc., Waltham, MA, United States), was performed using a 10% or 14% SDS-Tris-glycine-polyacrylamide gel. After electrophoretic separation, proteins were transferred to a PVDF membrane or to a nitrocellulose membrane (0.2 μm pore size) either for 90 min at 4°C and 100 V using a tank blot system or for 30 min at room temperature and 25 V using a semi-dry transfer system from BioRad (Trans-Blot^®^ Turbo Transfer System). Afterwards, membranes were incubated for 1 h in 3% skimmed milk in TBS-T to block unspecific binding sites and incubated over night at 4°C in primary antibody solutions (Table 2). After washing membranes for 30 min in TBS-T, they were incubated in secondary antibody solutions (Table 2) for 1 h and washed again with TBS-T. Proteins were detected using Amersham™ ECL™ Prime Western Blotting Detection Reagents (Merck Millipore, Burlington, MA, United States) (prepared 1:1) in a FUSION SL Vilber Lourmat (Peqlab) Gel Chemiluminescence Documentation System.

**TABLE 2:** List of antibodies used.

Target	Host-species	Order number
CD44 C-term	Rabbit	Abcam ab157107
CD44 primary conjungated	Mouse	Cell Signaling Technology #3516
ADAM10	Rabbit	Abcam ab1997
MMP14	Mouse	Millipore MAB3328-25UG
FLAG	Mouse	Sigma-Aldrich F1804
β-actin	Rabbit	Sigma-Aldrich A2066
Rabbit	Mouse	Santa Cruz Biotechnology, Inc. sc-2357
Mouse		Santa Cruz Biotechnology, Inc. sc-516102
Rabbit	Donkey	ThermoScientific A21206
Mouse	Donkey	ThermoScientific A21203

### 4.6 Immunofluorescent staining

#### 4.6.1 CD44 and meprin β

HeLa cells were seeded onto cover slips in a 12-well cell culture plate at a concentration of 2 × 10^5^ cells per ml. Medium was removed after 24 h and cells were washed three times with PBS, then incubated for 20 min at room temperature in PBS +4% PFA and washed three times with PBS again. Cells were permeabilized for 5 min in PBS +0.2% saponin and subsequently quenched for 10 min in PBS +0.12% glycin +0.2% saponin. After blocking in PBS +10% FBS +0.2% saponin for 1 h, cells were incubated over night at 4°C in primary antibodies dissolved in PBS +10% FCS +0.2% saponin. Cells were washed five times in PBS +0.2% saponin and incubated in secondary antibody solutions diluted 1:200 in PBS +10% FCS +0.2% saponin for 1 h at room temperature in the dark. Afterwards, cells were washed four times in PBS +0.2% saponin and twice in dH_2_O. Cover slips were mounted with Mowiol-DABCO (Boston BioProducts, Inc., Milford, MA, United States) and DAPI (Life Technologies, Carlsbad, CA, United States) (final concentration: 1 μg/mL). Images were taken at Olympus Fluoview 1000 CLSM.

#### 4.6.2 CD44 and F-aktin (Phalloidin)

HeLa cells were plated on 12 mm glas coverslips and fixed with 3.7% formaldehyde for 15 min At room temperature. Afterwards, the cells were washed twice with PBS-T (0.05% Tween-20) and incubated for 2 h with blocking solution (DPBS containing 5% NGS and 0.3% Tween-20). The cells were incubated over night with a primary CD44 antibody (abcam ab157107) diluted 1:100 in antibody dilution buffer (DPBS containing 1% BSA and 0.3% Tween-20) at 4°C in the fridge and stained with a fluorophore conjugated secondary antibody (α-rabbit Alexa Fluor 568) for 2 h at room temperature the next day. The coverslips were washed twice with PBS-T (0.05% Tween-20), stained for 1 h with Phalloidin and DAPI and mounted on an object slide with Mowiol.

### 4.7 FACS staining and analysis

HeLa cells were cultivated in 6-well plates and on the day of measurement detached using cold PBS containing 10 mM EDTA for 1 h. The cell suspension was collected in 1.5 mL reaction tubes and centrifuged for 5 min At 350 rcf. Afterwards, the cells were diluted in 100 µL FACS staining buffer (DPBS containing 1 mM EDTA and 0.5% BSA) and 1 µL of primary conjugated CD44 Alexa Fluor 488 (Cell Signaling Technology #3516) antibody was added. The cells were incubated with the antibody for 30 min at 4°C and occasional shaking and washed once with DPBS. Directly before the FACS measurement, the cell pellet was eluted in 100 µL DPBS and analyzed with a FACS Canto II from BD Bioscience. To determine the amount of background activity, an isotype control using an α-mouse IgG2B-Alexa Fluor 488 (R&D Systems) antibody was performed.

### 4.8 Scratch assay

HeLa cells were seeded into 12-Well cell culture plates in a final concentration of 1.5 × 10^5^ cells per well. After 24 h a 100 μL pipette tip was used to generate a scratch in the middle of each well. Cells were washed once with PBS afterwards to remove floating cells and medium was restocked. At the indicated time points, images were taken using a Keyence BZ-X810 microscope and analyzed using ImageJ.

### 4.9 Cell viability assay

HeLa cells were seeded in a 96-Well cell culture plate at a concentration of 2 × 10^4^ cells per ml and 200 µL per well. Cells were incubated for different time periods (24 h, 48 h and 72 h). To analyze cell viability, cell culture medium was changed to 3-(4,5-dimethylthiazol-2- yl)-2,5-diphenyltetrazolium bromide (AppliChem GmbH, Darmstadt, Germany) (MTT)-containing medium (0.5 mg/mL) and incubated for 4 h. Subsequently medium was discarded and remaining formazan crystals were resuspended in 200 μL isopropanol per well. Absorption was measured at 595 nm using a Tecan GENiosTM plate reader.

### 4.10 Organoid formation assay

HeLa cells were seeded into ultra-low adhesion 96-Well plates (Fisher Scientific GmbH, Schwerte, Germany) at a concentration of 1 × 10^4^ cells/mL at a volume of 200 µL per well. Images were taken after 4 days at a Keyence BZ-X810 microscope and images were analyzed using ImageJ.

### 4.11 Adhesion Plate and Wash assay

For cell adhesion assays 96-Well cell culture dishes were either coated with sodium hyaluronate (Fisher Scientific GmbH (ACROS Organics), Schwerte, Germany) at a concentration of 1 mg/mL in PBS or with 1% BSA in PBS as a control and incubated for 2 hours at room temperature. The plates were then washed once with PBS, blocked for 15 min at room temperature with 1% BSA in PBS, washed again with PBS and then air dried overnight under aseptic conditions at room temperature. A cell suspension of HeLa cells was prepared at a concentration of 5 × 10^5^ cells per ml and 100 μL were seeded into each well. Cells were cultivated for three different time periods (60, 30, 15 min). Afterwards cell culture medium was gently decanted and plates were washed twice in a PBS bath. Plates were then dried by gently hitting on a paper tissue. 50 μL of fix and stain solution, containing 0.1% crystal violet and 20% methanol diluted in dH_2_O, was added per well and incubated at 4°C overnight. Afterwards, cells were washed with dH_2_O to remove unspecific staining and dried by gently hitting on a paper tissue. Cells were permeabilized by adding 0.1% Triton X-100 in dH_2_O for 4 h at room temperature and absorption was measured at 595 nm using a Tecan GENiosTM plate reader.

### 4.12 Analysis of GTEx data

Gene Tissue Expression (GTEx) data was accessed through the Gepia2 server (http://gepia2.cancer-pku.cn/). Tissue expression data for all genes analysed was downloaded as reads per million. Calculations were performed in Excel before transfer into GraphPad Prism 9.

### 4.13 Statistical analysis

All graphs and statistical analyses were prepared using GraphPad Prism nine software. Data was analyzed by unpaired Student’s t-test, one-way ANOVA or two-way ANOVA. *p* < 0.05 was considered as statistically significant. Significance values were labelled in the following way: *p < 0.05, **p < 0.01, ***p < 0.001 ****p < 0.0001. N-numbers for individual experiments are given in figure legends.

## Data Availability

The original contributions presented in the study are included in the article/Supplementary Material, further inquiries can be directed to the corresponding author.
