# Supplementary material for: Proteolysis of CD44 at the cell surface controls a downstream protease network
Source: Front Mol Biosci. 2023 Feb 17;10:1026810. doi: 10.3389/fmolb.2023.1026810 (PMC9981664; doi:10.3389/fmolb.2023.1026810)
Supplement: Supplementary file 1 [file DataSheet1.PDF]

Supplemental Data:

Supplemental Figure 1:

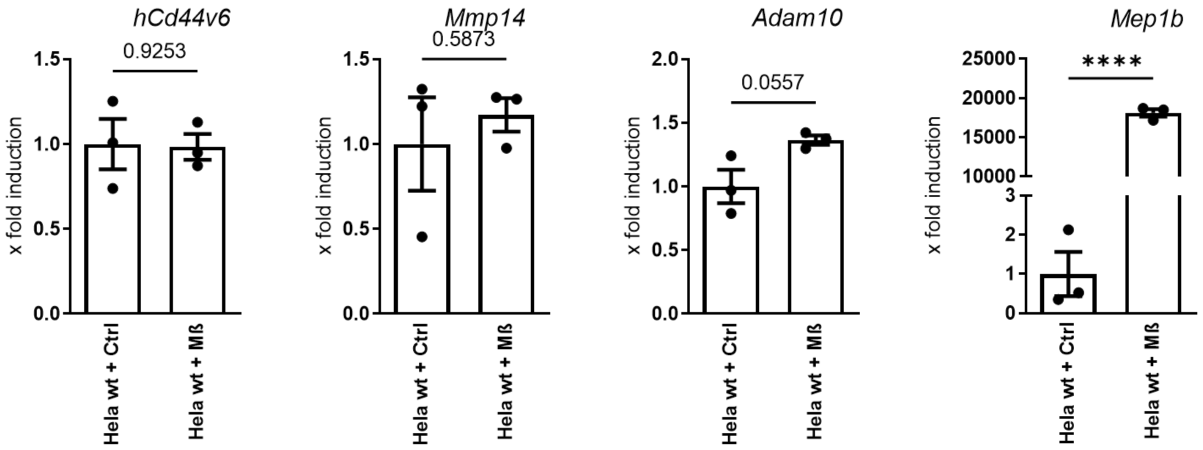

sFig1: Transcriptional regulation of CD44, ADAM10 and MMP14 after transient expression of meprin β in HeLa cells.

Supplemental Figure 2:

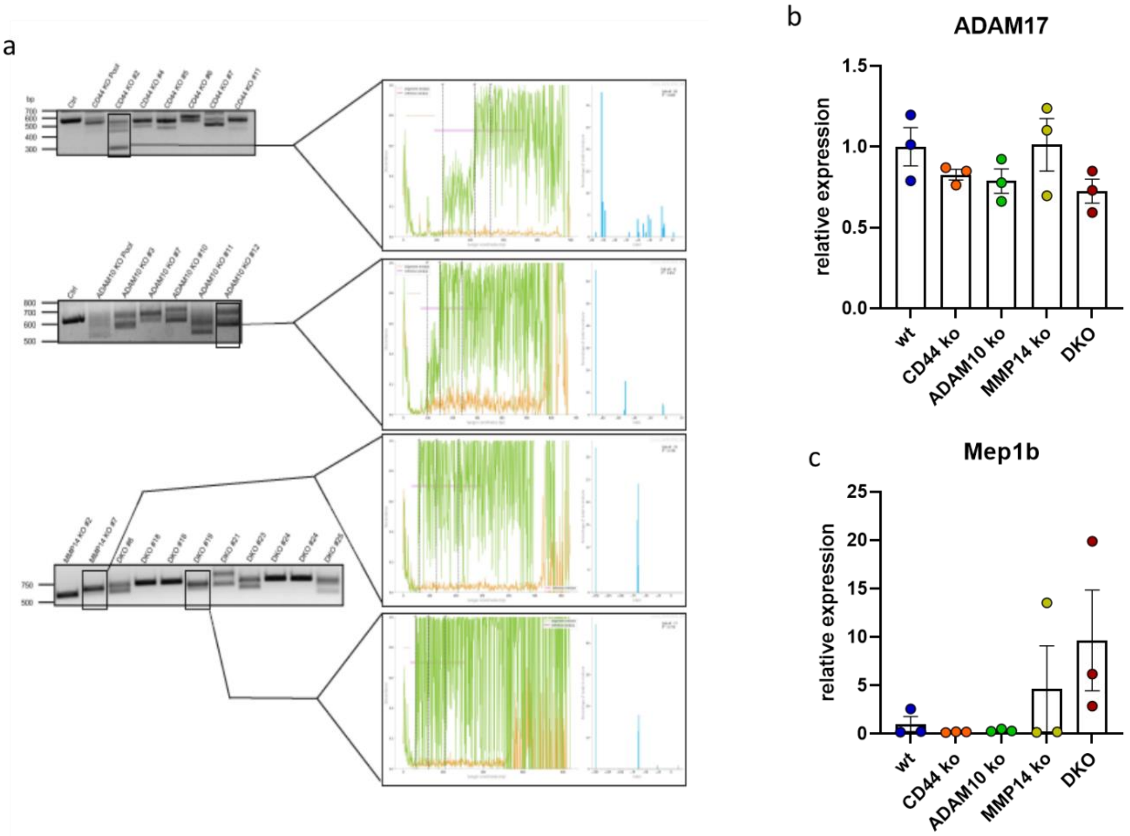

sFig 2: a) Agarose gels and sequencing results for the different CRISPR/Cas9 cell lines generated (upper row: CD44; middle row: ADAM10; lower row MMP14 and ADAM10/MMP14 double deficient (DKO) lines; Images obtained from Synthego Corporation, Redwood City, USA). b) Expression of ADAM17 in different CRISPR/Cas9 generated cell lines or parental HeLa cells. c) Expression of meprin β in different CRISPR/Cas9 generated cell lines or parental HeLa cells.

sTab 1: Complete statistical analysis for Fig. 4b

Within each row, compare columns (simple effects within rows)					
Number of families	3				
Number of comparisons per family	10				
Alpha	0,05				
Tukey's multiple comparisons test	Mean Diff,	95,00% CI of diff,	Below threshold?	Summary	Adjusted P Value
15 min					
wt vs. CD44 ko	7,78E-08	-0,05448 to 0,05448	No	ns	>0,9999
wt vs. ADAM10 ko	-2,22E-08	-0,09374 to 0,09374	No	ns	>0,9999
wt vs. MMP14 ko	-2,22E-08	-0,04660 to 0,04660	No	ns	>0,9999
wt vs. DKO	-3,33E-08	-0,07908 to 0,07908	No	ns	>0,9999
CD44 ko vs. ADAM10 ko	-1,00E-07	-0,07875 to 0,07875	No	ns	>0,9999
CD44 ko vs. MMP14 ko	-1,00E-07	-0,03214 to 0,03214	No	ns	>0,9999
CD44 ko vs. DKO	-1,11E-07	-0,05855 to 0,05855	No	ns	>0,9999
ADAM10 ko vs. MMP14 ko	0	-0,07294 to 0,07294	No	ns	>0,9999
ADAM10 ko vs. DKO	-1,11E-08	-0,06488 to 0,06488	No	ns	>0,9999
MMP14 ko vs. DKO	-1,11E-08	-0,06438 to 0,06438	No	ns	>0,9999
30 min					
wt vs. CD44 ko	0,07786	0,01731 to 0,1384	Yes	*	0,0136
wt vs. ADAM10 ko	-0,05435	-0,1613 to 0,05259	No	ns	0,4557
wt vs. MMP14 ko	-0,008174	-0,08635 to 0,07000	No	ns	0,9956
wt vs. DKO	0,009072	-0,07827 to 0,09641	No	ns	0,9957
CD44 ko vs. ADAM10 ko	-0,1322	-0,2648 to 0,0004155	No	ns	0,0507
CD44 ko vs. MMP14 ko	-0,08603	-0,2032 to 0,03112	No	ns	0,1742
CD44 ko vs. DKO	-0,06879	-0,1672 to 0,02959	No	ns	0,2046
ADAM10 ko vs. MMP14 ko	0,04617	-0,04970 to 0,1420	No	ns	0,502
ADAM10 ko vs. DKO	0,06342	-0,02537 to 0,1522	No	ns	0,191
MMP14 ko vs. DKO	0,01725	-0,06471 to 0,09920	No	ns	0,9441
60 min					
wt vs. CD44 ko	1,128	0,6002 to 1,655	Yes	***	0,0005
wt vs. ADAM10 ko	0,5364	0,07823 to 0,9945	Yes	*	0,0227
wt vs. MMP14 ko	1,205	0,6361 to 1,774	Yes	***	0,0006
wt vs. DKO	0,5671	-0,3561 to 1,490	No	ns	0,2974
CD44 ko vs. ADAM10 ko	-0,5914	-1,135 to -0,04803	Yes	*	0,0331
CD44 ko vs. MMP14 ko	0,07725	-0,2570 to 0,4115	No	ns	0,9239
CD44 ko vs. DKO	-0,5606	-1,716 to 0,5953	No	ns	0,496
ADAM10 ko vs. MMP14 ko	0,6686	0,2999 to 1,037	Yes	**	0,0016
ADAM10 ko vs. DKO	0,03079	-0,6387 to 0,7003	No	ns	0,9998
MMP14 ko vs. DKO	-0,6379	-1,618 to 0,3424	No	ns	0,254

sTab 2: Complete statistical analysis for Fig. 4d

Dunnett's multiple comparisons test	Mean Diff,	95,00% CI of diff,	Below threshold?	Summary	Adjusted P Value
0 h					
wt vs. CD44 ko	0				
wt vs. ADAM10 ko	0				
wt vs. MMP14 ko	0				
wt vs. DKO	0				
24 h					
wt vs. CD44 ko	18,26	4,319 to 32,20	Yes	**	0,0087
wt vs. ADAM10 ko	3,489	-13,17 to 20,14	No	ns	0,9507
wt vs. MMP14 ko	25,65	12,22 to 39,09	Yes	***	0,0004
wt vs. DKO	11,63	-2,609 to 25,86	No	ns	0,1307
48 h					
wt vs. CD44 ko	29,91	19,72 to 40,10	Yes	****	<0,0001
wt vs. ADAM10 ko	5,138	-6,710 to 16,99	No	ns	0,6157
wt vs. MMP14 ko	32,12	21,89 to 42,35	Yes	****	<0,0001
wt vs. DKO	12,44	1,979 to 22,90	Yes	*	0,0164
72 h					
wt vs. CD44 ko	20,57	12,89 to 28,26	Yes	****	<0,0001
wt vs. ADAM10 ko	2,889	-2,588 to 8,367	No	ns	0,4077
wt vs. MMP14 ko	14,83	6,249 to 23,42	Yes	**	0,0018
wt vs. DKO	1,612	-0,2437 to 3,467	No	ns	0,0985
